# Cerebrospinal fluid dynamics in non-acute post-traumatic ventriculomegaly

**DOI:** 10.1186/s12987-020-00184-6

**Published:** 2020-03-30

**Authors:** Afroditi D. Lalou, Virginia Levrini, Marek Czosnyka, Laurent Gergelé, Matthew Garnett, Angelos Kolias, Peter J. Hutchinson, Zofia Czosnyka

**Affiliations:** 1 Division of Neurosurgery, Department of Clinical Neurosciences, University of Cambridge and Cambridge University Hospital NHS Foundation Trust, Cambridge, UK; 2 Department of Intensive Care, Hôpital privé de la Loire, Saint Etienne, France

**Keywords:** Cerebrospinal fluid, CSF dynamics, CSF infusion test, Hydrocephalus, Traumatic brain injury, Ventriculomegaly

## Abstract

**Background:**

Post-traumatic hydrocephalus (PTH) is potentially under-diagnosed and under-treated, generating the need for a more efficient diagnostic tool. We aim to report CSF dynamics of patients with post-traumatic ventriculomegaly.

**Materials and methods:**

We retrospectively analysed post-traumatic brain injury (TBI) patients with ventriculomegaly who had undergone a CSF infusion test. We calculated the resistance to CSF outflow (Rout), AMP (pulse amplitude of intracranial pressure, ICP), dAMP (AMPplateau-AMPbaseline) and compensatory reserve index correlation coefficient between ICP and AMP (RAP). To avoid confounding factors, included patients had to be non-decompressed or with cranioplasty > 1 month previously and Rout > 6 mmHg/min/ml. Compliance was assessed using the elasticity coefficient. We also compared infusion-tested TBI patients selected for shunting versus those not selected for shunting (consultant decision based on clinical and radiological assessment and the infusion results). Finally, we used data from a group of shunted idiopathic Normal Pressure Hydrocephalus (iNPH) patients for comparison.

**Results:**

Group A consisted of 36 patients with post-traumatic ventriculomegaly and Group B of 45 iNPH shunt responders. AMP and dAMP were significantly lower in Group A than B (0.55 ± 0.39 vs 1.02 ± 0.72; p < 0.01 and 1.58 ± 1.21 vs 2.76 ± 1.5; p < 0.01. RAP baseline was not significantly different between the two. Elasticity was higher than the normal limit in all groups (average 0.18 1/ml). Significantly higher Rout was present in those with probable PTH selected for shunting compared with unshunted. Mild/moderate hydrocephalus, ex-vacuo ventriculomegaly/encephalomalacia were inconsistently reported in PTH patients.

**Conclusions:**

Rout and AMP were significantly lower in PTH compared to iNPH and did not always reflect the degree of hydrocephalus or atrophy reported on CT/MRI. Compliance appears reduced in PTH.

## Background

Post-traumatic hydrocephalus (PTH) is potentially under-diagnosed and under-treated, creating the need for a more efficient diagnostic tool [1–3]. Currently, PTH is diagnosed using a combination of clinical assessment and brain imaging. By nature of the vast and varied sequelae of traumatic brain injury (TBI), clinical signs and symptoms are variable and difficult to identify consistently. The need to distinguish between ventriculomegaly secondary to PTH versus brain atrophy by imaging techniques, poses a further challenge to diagnosis [4–6]. Also, different forms of PTH can be classified according to the phase after injury. In the first few days to weeks, there may be obstruction of normal pathways to CSF flow manifested by enlarged ventricles and raised ICP. This is acute hydrocephalus and frequently requires an external ventricular drain (EVD). An alternative form of acute hydrocephalus, without ventricular enlargement, is ‘external hydrocephalus’ due to impairment of CSF absorption in Pacchionian granulations [1, 2] when only the cranial subarachnoid space is enlarged. Different trials, especially recent decompressive craniectomy trials, have reported variable incidences of hydrocephalus post severe TBI, and the rate of reporting hydrocephalus as a complication of TBI varies between 0.7 and 50% [7–11].

In the late phase after TBI, patients can present with symptoms or signs similar to idiopathic normal pressure hydrocephalus (iNPH) resulting from impairment of CSF circulation in the subarachnoid space in response to the post-traumatic inflammatory process. The ventricles are enlarged but ICP remains normal [1–3, 12]. Post-acute PTH could inhibit rehabilitation and be the main contributing factor to poor long-term outcome after TBI [13, 14]. Measurements of opening pressure via lumbar puncture and spinal tap test are often used to detect PTH and select patients for shunting but are not diagnostically accurate [3, 12, 15–18]. Since ICP is usually normal in chronic PTH, we hypothesize that resistance to CSF outflow (Rout) could be abnormal. Nonetheless, short-term manometric assessment via lumbar puncture is still the standard practice in neurosurgery [19, 20].

Various reports of post-traumatic hydrocephalus exist, and attempts have been made to identify risk factors for PTH, including age, presence of subarachnoid bleeding [13, 21] and size and number of decompressive craniectomies [13, 14, 21]. Infusion test parameters, such as resistance to CSF outflow (Rout), CSF pulse amplitude (AMP) and compensatory reserve index RAP, have been extensively reported before in hydrocephalus, normal subjects, and post-TBI [1, 12, 19, 21–24]. Rout, derived from the rise of ICP during infusion compared to the baseline ICP has been utilised and trialed in iNPH and NPH of mixed aetiology to guide shunting decisions, however, it has not been investigated sufficiently in PTH cohorts for this purpose. Marmarou et al. [1] described how CSF dynamics can aid in the detection of post-traumatic hydrocephalus. They used the bolus-injection method, to calculate Rout in patients within 3 months of their traumatic brain injury and classified patients into 5 groups: (1) normal (no ventriculomegaly + ICP ≤ 15 mmHg), (2) benign intracranial hypertension (no ventriculomegaly + ICP > 15 mmHg), (3) atrophy (ventriculomegaly + ICP ≤ 15 mmHg + Rout ≤ 6 mmHg/min/ml, (4) NPH (ventriculomegaly + ICP ≤ 15 mmHg +Rout > 6 and, (5) high-pressure hydrocephalus (ventriculomegaly + ICP > 15 mmHg) [1]. They proposed an opening ICP higher than 15 mmHg or Rout higher than 6 mmHg/ml/min as potential thresholds for shunting. This bolus injection-derived Rout, however, may be significantly lower than one calculated from constant-rate infusion and the other infusion methods [25, 26]. To best knowledge, other infusion test parameters besides ICP and Rout have not been studied in PTH.

Long recordings (ideally several hours) of baseline ICP and CSF dynamics required in order to properly estimate CSF parameters and ICP monitoring are quite invasive [1, 16, 19, 26–28]. Instead, our department has been using constant-rate infusion studies for patients with hydrocephalus and other CSF disorders. In this paper, we report our experience and discuss the utility of CSF infusion testing in patients with post-traumatic ventriculomegaly. More specifically, with constant-rate infusion tests, we have analysed CSF dynamics in post-acute, post-traumatic ventriculomegaly with normal baseline ICP. We measured parameters such as AMP, RAP and the response to infusion, currently lacking in the literature for PTH. Lastly, we compared TBI patients to a group of iNPH shunt-responder patients, to determine if the classic threshold of Rout 13 mmHg/min/ml as well as other known CSF dynamics thresholds for iNPH apply to PTH.

## Methods

### Patient selection

We retrospectively identified patients from our infusion study database at Cambridge University Hospital NHS Foundation Trust who had a background of TBI. The type of TBI varied widely amongst subjects in severity (mild-severe), time interval since injury and type of injury (subdural haematoma, subarachnoid haemorrhage, contusion). All patients underwent an infusion study following request from a Consultant Neurosurgeon with a subspecialty interest in neurotrauma or hydrocephalus. There are no local or national guidelines specifying criteria for performing infusion tests in TBI patients and therefore practice amongst consultants could have been variable. However, these were all patients with ventriculomegaly on CT or MRI (as reported by a consultant neuroradiologist) and with clinical features of PTH.

Our inclusion criteria were as follows:

- Tests were performed between January 2011 and February 2019. We started at 2011 in order to ensure better access to clinical notes and homogeneity in consultant neurosurgeons, neuroradiologists and radiology reports.
- No missing bone flap at the time of the test. This is because decompressive craniectomy (DC) has significant effects on pressure–volume compensation.
- If DC had been previously performed, a cranioplasty should have been performed 4 weeks or more before the infusion to allow for restoration of the intracranial circulation (CSF as well as cerebral blood flow) [3].
- Rout > 6 mmHg/min/ml without possible high degree of atrophy from the CT/MRI as reported by a neuroradiologist, and baseline ICP < 15 mmHg because these patients may have high pressure hydrocephalus, intracranial hypotension or brain atrophy (as defined in the background section), with altered CSF dynamics [3].

Finally, we used a comparison group of consecutive, gender-matched idiopathic Normal Pressure Hydrocephalus (iNPH) patients with ventriculomegaly and clinical symptoms suggestive of NPH, that had a lumbar infusion test as part of their routine investigations and a positive response to shunt surgery with clinical documentation of improved symptoms at 6-month follow-up. The iNPH group had undergone infusion studies between 2003 and 2018 and the results previously reported [29–31]. Normal controls were not available, as all studies in our centre are performed on clinical indication.

### Infusion test

Infusion studies were carried out via lumbar puncture (LP) with the patient in the lateral decubitus position or via Ommaya reservoir with the patient supine. Data was collected via connection of a fluid-filled pressure transducer (Edwards Lifesciences™, Irvine, USA) and pressure amplifier (Spiegelberg, Hamburg, Germany or Philips, Amsterdam, The Netherlands) to either the 18-gauge LP needle or two 25-gauge butterfly needles respectively. Following ten minutes recording of baseline ICP, Hartmann’s solution was infused at a constant rate of 1.5 ml/min and recording was continued for a further 10 min after ICP had reached its plateau. Data was processed using ICM + software (University of Cambridge Enterprise Ltd) and saved in our infusion study database. Our constant infusion method and analysis has been described in previous publications [32–36]. Appropriate consent, in line with national guidance, was in place for the procedure described and for use of their data for research purposes.

### Data collection and analysis

The following CSF dynamics parameters were extracted from our database: ICP baseline (ICPb), ICP at plateau (ICPp), resistance to outflow (Rout), and fundamental amplitude of ICP pulse (AMP). Pressure–volume compensation and compliance data were collected as the compensatory reserve index RAP, slope of AMP-ICP line (AMP-P) and Elasticity. RAP is calculated as the moving correlation coefficient between ICP and AMP. A high correlation (> 0.6) has been described as indicative of disturbed pressure–volume relationship, indicating reduced compensatory reserve [37]. Elasticity is a compliance index of the brain, with values > 0.18/ml associated with poor compliance [38–40]. The slope of the AMP-P line is a descriptor of both the elasticity and the cerebral blood volume (AMP-P slope = elasticity * cerebral blood volume delivered in each cardiac cycle) [41].

Additional clinical data: patient demographics, date/severity of TBI, date of infusion study, cranioplasty date (if applicable) and brain imaging, was extracted from the hospital electronic health record system. CT and MRI scans performed closest to the time of the infusion study (within 3 months before or after) were independently analysed by co-authors ADL and VL. The frontal horn width (FHW), frontal occipital horn ratio (FOHR) and Evan’s ratio were measured on 28 CT and 6 MRI scans (scans for 4 subjects were not available). We used the one-sample Wilcoxon test to demonstrate the difference between normal ventricular indices values and the means for our possible PTH cohort: 0.3 for Evan’s, 0.4 for FOHR and 39 mm for FHW [42]. Volumetric analysis was not possible, as MRI scans were only available for six patients.

Statistical analysis was carried out using R software version 3.5.2. Comparisons between groups were tested using non-parametric tests, mainly the Mann–Whitney U test for independent samples. We used the Kruskal-Wallis test followed by pairwise wilcoxon test in order to compare differences among 3 groups. We have used Pearson’s or Spearman’s correlation when appropriate, depending on how much our data deviated from a bivariate normal distribution or an asymptotically normal distribution. P-values of less than 0.05 were considered statistically significant.

## Results

### Patient population and classification

**Fig. 1:**
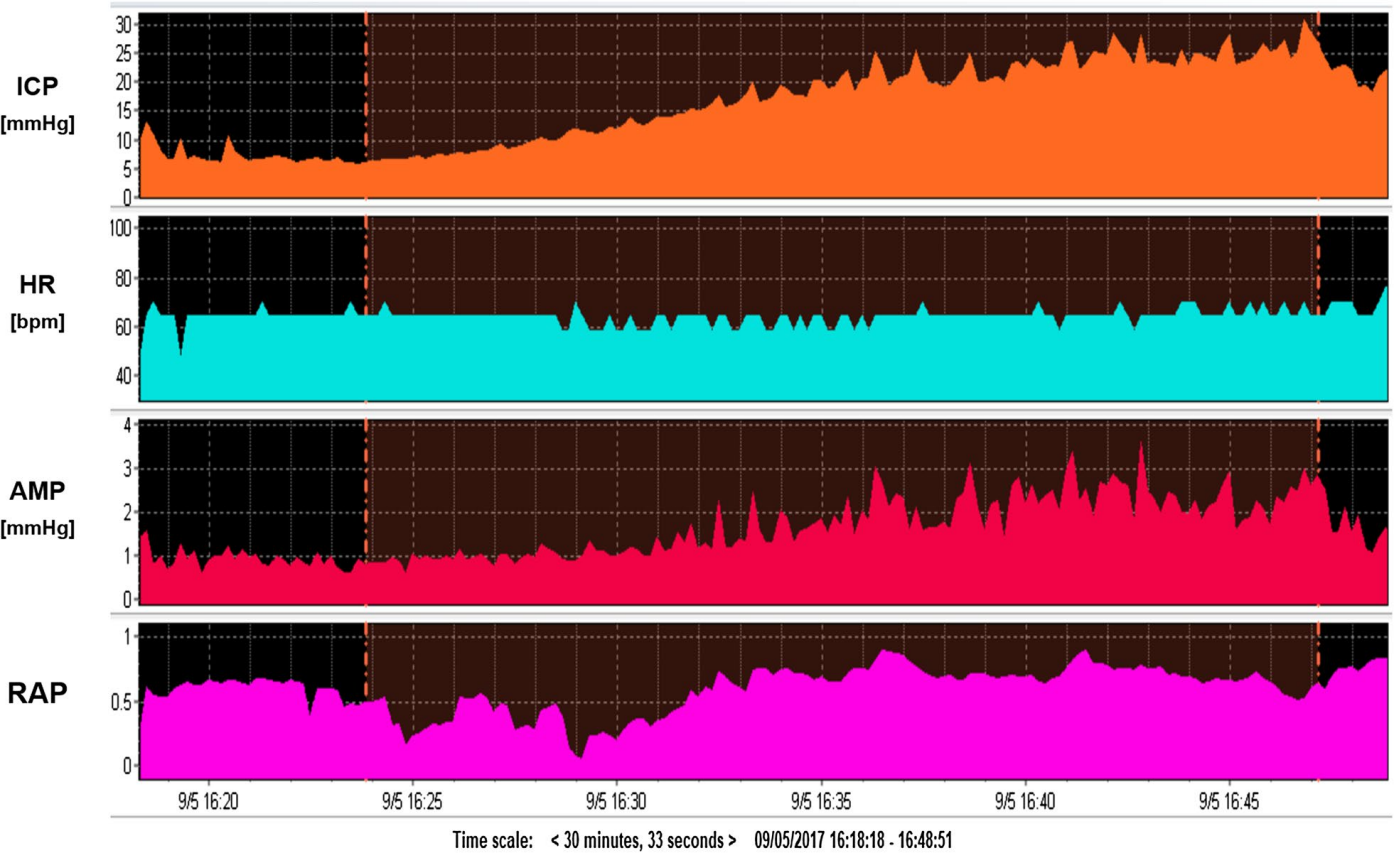
Representative example of CSF dynamics in a patient under investigation for possible Post Traumatic Hydrocephalus. ICP (monitored via Ommaya reservoir in this case) increased briskly after start of infusion, with an Rout around 11–13 mmHg/min/ml. AMP at baseline ~ 1 mmHg, also reacted briskly to infusion until a plateau of 5.6 mmHg. RAP at baseline ~ 0.6, clearly increased to almost 1 after infusion of only a few ml, indicating exhaustion of compensatory reserve. CSFp: CSF pressure (access to the CSF space via LP). AMP: fundamental amplitude of ICP. RAP: compensatory reserve index (moving correlation coefficient between ICP and AMP)

An initial search of the database found a total of 46 infusion tests were carried out on 44 TBI patients during the defined time period and 36 (12 females and 24 males) matched our inclusion criteria and were assigned to group A (the ‘possible PTH’ group). The time interval between the TBI and infusion varied between subjects, from 10 days to a maximum of 33.5 years. The TBI date for 11 subjects could not be retrieved and of the remaining 25, the average time interval was 56 months. From the records of the 25 patients, 19 had been initially classified as having ‘severe’ and 6 ‘mild’ TBI according to Glasgow Coma Scale. From the 36 included patients, average age 53 ± 17 years, 26 required a LP for CSF space access, whereas 10 had a pre-implanted Ommaya reservoir, 24 had an intact cranial vault and 8 had a cranioplasty in situ. All 36 patients had Rout, AMP, rise in AMP during infusion compared to baseline (dAMP) and RAP both at baseline and at plateau, as shown in Table 1. A representative example of an infusion test performed on a patient under investigation for post-traumatic hydrocephalus is shown in Fig. 1.

**Table 1:** Comparison of CSF dynamics in Groups A (Post traumatic hydrocephalus) versus B (possible iNPH who positively responded to shunt surgery)

Mean	Group A (N = 36)	Group B (N = 45)	p-value
ICPb [mmHg]	9.31 ± 4.12	9.48 ± 4.57	ns
Rout [mmHg/min/ml]	13.53 ± 5.21	19 ± 8.91	p < 0.001
AMPb [mmHg]	0.55 ± 0.39	1.02 ± 0.72	p < 0.05
dAMP [mmHg]	1.58 ± 1.21	2.76 ± 1.50	p < 0.001
Slow [mmHg]	0.66 ± 0.68	1.26 ± 1.5	ns
AMP-P slope	0.09 ± 0.05	0.14 ± 0.08	p < 0.05
Elasticity [1/ml]	0.19 ± 0.13	0.19 ± 0.1	ns
RAPb	0.57 ± 0.18	0.38 ± 0.21	ns
RAPinf	0.95 ± 0.07	0.92 ± 0.075	ns

Results are shown as mean ± SD. ICPb: Intracranial pressure at baseline. Rout: resistance to out flow. AMP: fundamental pulse amplitude of ICP. dAMP: AMP plateau—AMP baseline. Slow: magnitude of slow waves of ICP. AMP-P slope: slope of the line derived from ICP-AMP linear regression. Elasticity: [1/ml]. RAPb: compensatory reserve index (moving correlation coefficient between ICP and AMP) at baseline. RAPinf: RAP during infusion

*ns* not significant

Group B included 45 iNPH patients who had undergone CSF infusion tests prior to shunting and had positively responded to ventriculoperitoneal shunting. The average age was 66.16 ± 12.80 years and was composed of 19 females and 26 males. Numerical results for CSF dynamics comparison between group A and B are found in Table 1, showing significantly lower Rout, AMPb and dAMP in group A compared to group B. The mean age differed significantly between the two groups (p < 0.01).

### Follow-up

After completing all assessments for PTH, 16 of the 36 patients in group A underwent insertion of a ventriculoperitoneal shunt. The decision was made by a consultant following assessment of symptoms, comorbidities, risks and benefits, and may have been partially influenced by a higher than normal Rout (using the traditional threshold of Rout > 13). At a 3-to-6 month follow-up, there were 5 cases with documented clinical improvement by the clinician, who also took family and/or rehabilitation facility reports into account. Complications post-shunting were documented in three cases: one case of haemorrhage, one infection and one shunt malfunction.

The clinicians responsible for 13 of the 36 patients decided against shunting following the above assessments. Finally, 7 of 36 cases were lost to follow-up, as there was no further documentation of procedures or clinic visits in their files. Results comparing shunted versus non-shunted patients with PTH, shunted PTH patients with group B and non shunted PTH with group B are shown in Table 2. Rout was significantly higher in iNPH and shunted PTH patients compared to non-shunted PTH patients.

**Table 2:** Comparison between shunted (n = 16) versus non-shunted (n = 13) PTH patients (7/36 were lost in follow-up), shunted PTH patients versus iNPH shunt responders (B group) and non-shunted PTH versus iNPH responders

Mean	Shunted (N = 16)	No shunt (N = 13)	p-value (shunt vs no shunt)	iNPH (Group B)	p-value (Shunt vs B)	p-value (no shunt vs B)
ICPb [mmHg]	8.74 ± 4.32	9.91 ± 3.6	ns	9.48 ± 4.57	ns	ns
Rout [mmHg/(min/ml)]	16.73 ± 5.67	10.56 ± 3.06	p < 0.01	19 ± 8.91	p < 0.001	p < 0.001
AMPb [mmHg]	0.54 ± 0.40	0.59 ± 0.43	ns	1.02 ± 0.72	p < 0.05	p < 0.05
dAMP [mmHg]	1.94 ± 1.64	1.35 ± 0.66	ns	2.76 ± 1.50	ns	p < 0.05
Slow [mmHg]	0.76 ± 0.7	0.45 ± 0.72	ns	1.26 ± 1.5	ns	ns
AMP-P slope	0.11 ± 0.06	0.066 ± 0.03	ns	0.14 ± 0.08	ns	p < 0.05
Elasticity [1/ml]	0.19 ± 0.11	0.2 ± 0.11	ns	0.19 ± 0.1	ns	ns
RAPb	0.6 ± 0.16	0.54 ± 0.2	ns	0.38 ± 0.21	ns	ns

*ns* not significant

### Relationship with imaging

Encephalomalacia or ex vacuo ventriculomegaly was evident in 12 of the 36 cases in Group A (possible PTH) and in 1 of the 16 shunted patients, whose condition was reported as unchanged post shunting. Only 3 of the 36 patients had a clear neuroradiologists’ reporting of a mild-moderate degree of hydrocephalus, two of whom were shunted. This shows disparity between the neuroradiologist’s reports and the CSF dynamic results.

### Ventricular indices and CSF dynamics

Numerical results for frontal horn width, fronto-occipital horn ratio and Evan’s ratio for PTH patients are shown in Table 3. Both groups had significantly different values from normal for all three measurements and the measurements were not significant between shunted and not shunted subgroups. There was no significant correlation or tendency for a strong correlation between ventricular measurements and any of the CSF dynamic parameters reported in the 34 patients of group A.

**Table 3:** Linear indices of ventricular size in our cohort of 34 patients with possible PTH (in two imaging was not available) and in those selected for shunting versus those not selected for shunting

Ventricular index	Group A (N = 34)	p-value (from norm)	Shunted (N = 16)	p-value (from norm)	Not shunted (N = 13)	p-value (from norm)	p-value (shunt vs no shunt)
FHW (mm)	50.30 ± 10.14	p < 0.001	52.46 ± 10.93	p < 0.001	47.50 ± 10.6	p < 0.05	ns
FOHR	0.47 ± 0.06	p < 0.001	0.48 ± 0.07	p < 0.01	0.45 ± 0.06	p < 0.01	ns
Evan’s	0.38 ± 0.07	p < 0.001	0.39 ± 0.08	p < 0.001	0.36 ± 0.07	p < 0.01	ns

Patients lost in follow-up were 5/34. FHW: frontal horn width. FOHR: frontal occipital horn ratio, Evan’s: Evans ratio. Normal values used were 39 mm for FHW, 0.4 for FOHR and 0.3 for Evan’s Index

*ns* not significant

## Discussion

We have investigated the utility of infusion studies for investigation of possible PTH and in comparison with infusion studies for iNPH.

### Comparison between TBI and iNPH groups

In selecting patients for Group A, we had to exclude those with decompressive craniectomy, and recent cranioplasty, as these radically influence CSF dynamics [3, 26–28], and are inconsistent with CSF dynamics in patients with an intact skull. We had also pre-specified the exclusion of patients with brain atrophy, as determined by Rout < 6 mmHg/min/ml [1, 43].

### Inclusion and exclusion criteria

1. The exclusion criteria of ICP > 15 mmHg was based on the rationale that this would be considered high-pressure hydrocephalus and would negate a meaningful comparison to our iNPH group. In reality, we did not have to exclude any patients due to this, as all had ICP < 15. If ICP is high in a single manometry test, patients would not usually be referred for an infusion test.
2. Rout < 6 mmHg/(ml/min) Physiological outflow resistance is around 7 mmHg/min/ml, therefore anything below that is not only inconsistent with hydrocephalus but also approaching pathologically low levels suggestive of atrophy or another pathology [44].

Comparing CSF dynamics in our probable PTH group with a control group is more informative than simply reporting results of the CSF dynamics in the PTH group alone. Unfortunately, there is a lack of data regarding normal CSF dynamics in healthy subjects which is why we selected a group of NPH shunt-responders for comparison. Our choice of iNPH shunt-responders as the comparison group was based on two points. On the one hand, iNPH group could highlight CSF disturbance patterns that may benefit from shunting so provide a meaningful group for comparison. On the other hand, it is also beneficial to highlight the differences between these two groups as known shunting thresholds for NPH may not be applicable to PTH.

The average baseline ICP did not differ significantly between patients tested for PTH and those with iNPH (Table 1). In contrast, pulse amplitude descriptors (AMP and dAMP) were significantly lower in possible PTH compared to iNPH. Due to a direct and strong correlation between AMP and ICP during infusion, it seems unexpected that, even though there was no difference in ICPb AMPb in particular was significantly lower in PTH than in iNPH. It appears that both groups approached an average RAP of 0.6, revealing depleted compensatory reserve in both primary NPH and NPH secondary to trauma [45–48], where a “healthy” compliant brain is considered RAP < 0.5. Elasticity also appeared increased in both PTH and iNPH groups compared to in health (where elasticity < 0.18 1/ml), implying decreased cerebral compliance. On the other hand, the slope of the AMP-P line was significantly lower in the possible PTH group, Table 1. Given that this slope has been described to correlate with elasticity, with increased slope correlating with increased elasticity and therefore decreased brain compliance, this finding is contradictory. However, since this group was not a “clean” population of PTH, but possible PTH, the influence of low AMP-P slope could appear numerically stronger in this preliminary cohort. Alternatively, since the relationship between the AMP-P slope is Elasticity multiplied by cerebral blood volume, a decreased cerebral blood volume could be linked to PTH and explain the low AMP-P slope and perhaps disturbance in CSF dynamics [12, 22, 49]. Rout in iNPH was on average lower than possible PTH, Table 1.

Of note, the comparison between our possible PTH group with an unknown shunt response and shunt-responsive iNPH was not aimed at comparing the two aetiologies and pathophysiological processes, but to utilise a group of CSF dynamics that clearly reflect a clinical diagnosis (confirmed iNPH). Similarly, iNPH shunt responders on average had a higher Rout than shunted PTH patients, Table 2. Although our samples were not large enough to definitively confirm such a difference, it is likely that the threshold of Rout for shunting in PTH could be lower than in iNPH and such a relationship should be examined further. On the other hand, the very few shunt responders we managed to report also had a higher Rout (average 18.86 ± 5.13 mmHg/min/ml). In a few cases, imaging reported both signs of hydrocephalus as well as an area of encephalomalacia. Due to the heterogeneity of TBI, it is possible that some patients will have areas of encephalomalacia secondary to the trauma, as well as impaired CSF reabsorption and vascular bed dysfunction, resulting in group A’s average Rout being lower than in shunt-responsive iNPH. Unfortunately, no reports were available on the degree of atrophy in the different patients, and we did not possess enough patients or the right imaging sequence in order to be able to quantify volume loss. The influence of encephalomalacia and different degrees of atrophy on CSF dynamics and Rout should be clarified through appropriately powered, randomised studies. Another contributing factor to the difference in Rout seen between the two groups could be the age of their populations, which differed significantly. A numerical, age-correction formula for Rout is not yet known, however there is evidence to suggest that Rout increases with age [50–53]. The significantly lower AMP in group A points towards cerebrovascular bed dysfunction [45–47, 54, 55] or decreased intracranial compliance. Again, this contributes to the argument against use of imaging alone for the identification of PTH.

Finally, since vascular bed reactivity can be altered following TBI, and some of our current findings are suggesting cerebral blood volume disturbance in PTH, it would be of interest to explore the cerebral blood flow autoregulation of these patients and how it relates to CSF circulation. Unlike “pure” iNPH and PTH shunt responders with high Rout, cerebral autoregulation and blood flow could vary in patients after TBI, especially severe TBI [3, 27, 30]. An inverse relationship between disturbed autoregulation and Rout has previously been reported in a mixed aetiology NPH cohort [30]. Our patients did not have the required parameters monitored in order to test this. Furthermore, previous decompressive craniectomy, especially if a reconstruction is delayed significantly, can have negative effects on brain perfusion and contribute to the development of ventriculomegaly [28]. We have not been able to explore here the effect of TBI severity on the development of PTH due to insufficient numbers of patients. Similarly, we could not explore the implication of previous decompressive craniectomy, neither the impact of length of stay without cranioplasty [3].

Comparisons of the lumbar and ventricular approaches to infusion testing are few as tests are usually performed (and compared) in different patients and the selection of patients is never blinded. A study by Borgensen et al. [36] attempted to answer the question and they found very good correlation and no difference between Rout calculated using lumbo-ventricular perfusion and lumbar infusion test. Later, the same group concluded that intraventricular infusion and lumbar infusion led to the same useful clinical conclusions [56]. Modern, randomised trials would be best to elucidate this question.

### Shunt surgery

Average Rout for group B was 19.00 compared to 16.69 in the 16 patients selected for shunting in group A, Table 2. However, the 5/16 shunted TBI patients with clearly documented improvement post-shunting had average Rout of 18.86 ± 5.13 (data not shown). Despite Rout being a contributing, but not the only, factor to the decision whether to insert a shunt, TBI patients that underwent shunting still had lower Rout compared to iNPH. We did not have an adequate number of shunt responders on follow-up to compare with the 45 iNPH shunt responders. Currently we cannot validate a threshold Rout value for shunting in TBI due to the small number of cases with follow-up available, as discussed in the limitations section. Preliminarily, we suggest that interpretation of Rout be made in association with good quality imaging in cases where elements of both PTH and encephalomalacia are present. On the contrary to Rout, slow waves of ICP did not seem to significantly differ between the two groups, despite a possible positive correlation between Rout and slow waves previously described [57]. Physiological and pathophysiological thresholds for b waves however are yet to be described and further work of their role in NPH and hydrocephalus is in progress.

If decreased compliance (or high Rout) is indeed a characteristic of PTH, this could not be demonstrated in our shunted versus not shunted patients. AMP, slow waves, and volume-pressure relationship descriptors did not differ between the shunted and non-shunted (perceived as normal) patients. This poses the question of whether a “stiffer” brain with limited compensatory space, as was the case in even our non-shunted group, really could be considered “normal”, or if they could be descriptors of unidentified hydrocephalus. As a result, in patients where compliance is poor, minimal disturbances of the intracranial pressure–volume relationship could be resulting in a clinical picture suspicious for PTH, justifying why these patients were initially tested, regardless of whether they were selected for shunting. Careful follow-up of all those patients regardless of shunting, perhaps with a prospective, long-term follow-up study, could assist us in determining the optimal multi-dimensional diagnostic pathway and also clarify whether quantitative analysis of CSF dynamics could contribute to such a pathway. Since infusion tests provide an objective and easily performed test in order to identify PTH, patients post TBI with ventriculomegaly could safely undergo testing routinely, at a timing that should preferably be soon after TBI [13, 14]. Timing in our dataset varied from months to years, however risk stratification, combined with strategic testing and building of better evidence should aim at drawing a tighter timeframe. The safety of the lumbar infusion test has been shown before in a large series of patients [18].

### Clinical implications

The fact that there is no high grade evidence for using infusion tests for patients with PTH was one reason for us to study the subject, since no one has attempted to build the evidence since Marmarou. Available methods and tools do not yet provide a reliable mean for expert neurosurgeons to base their decision to shunt. There is currently no proven, readily available and cheap investigation for PTH. Volumetric studies with flow MRI and high definition MRI have also not yet been validated in clinical trials. Objective testing of CSF dynamics via infusion tests could potentially differentiate between atrophy and non-acute hydrocephalus and contribute in shaping firm diagnostic criteria for PTH and subsequently assessing the incidence of PTH. It is a safe procedure, with infection being a complication observed in < 1% of performed tests [18]. As under diagnosis and under treatment is possible in non-acute PTH, infusion tests could serve as both a screening and a diagnostic tool.

### Imaging PTH

From our results we can conclude that no linear ventricular metric can be associated with baseline CSF pressure or any other CSF circulation and pressure-volume compensation in PTH and that linear ventricular indices also could not be used in selecting patients for shunting. We could only use those indices to underpin ventriculomegaly and therefore select patients for further testing. The lack of reliability or of diagnostic and predictive value of such measures is now well-established both in the radiology and NPH fields and are becoming obsolete [42, 58, 59]. Hydrocephalus was reported as mild/moderate/severe by the neuroradiologists, however the result of the infusion did not match those descriptions, i.e. some patients with mild hydrocephalus showed disturbed CSF dynamics with significantly high Rout, whereas patients described as atrophic actually had significant disturbance of their CSF dynamics. Of note, there was one case where the neuroradiologist reported solely several areas of encephalomalacia, however CSF dynamics were consistent with hydrocephalus.

### PTH with normal baseline pressure—underdiagnosed?

Post-traumatic hydrocephalus has generally been reported to an incidence of 10–40% and difficult entities such as our currently explored PTH group with normal pressure pose significant diagnostic challenges. We saw that the small number of recruited patients within a long period of time (7 years) that there has been a lack of standardization of when to test and perhaps even underestimation of the incidence of the disease, its natural progression and the importance of closer, objective monitoring of all cases of ventriculomegaly.

### Limitations for this study

We selected a heterogenous group of TBI patients, with different types of injuries. Furthermore, the time frame post-TBI varied from weeks to years and in some cases, the exact date of the TBI was not available. As the time delay of untreated chronic hydrocephalus impacts the likelihood of improvement post-shunting, the heterogeneity of our sample may have impacted our ability to interpret improvement post-shunting in the ‘likely PTH’ group. In addition, we have not been able to perform a detailed analysis of brain imaging in our patients and most of them were subjected to routine CT scans, which limited the available information. Our results were further limited to some patients being lost to follow-up.

Finally, the infusion studies in our database were done either under local anaesthetic or general anaesthetic (GA). There is currently no definitive data demonstrating the effects of GA on compensatory reserve and CSF dynamics, other than a reduction of the magnitude of B waves during general anaesthesia. In Lalou et al. [29], we reported a significant difference in Rout between awake and GA patients, however this could have been due to a selection bias (patients with more severe symptoms and a higher clinical suspicion underwent infusion tests under GA immediately before shunting). No other parameters were influenced by GA.

## Conclusions

Rout and AMP were significantly lower in PTH compared to iNPH and did not always reflect the degree of hydrocephalus or atrophy reported on CT/MRI. Compliance appears reduced in PTH.

With regards to CSF dynamics, more studies are needed in order to assess PTH versus normal CSF circulation, and shunt responders vs non-responders, as well as to quantify thresholds for Rout and/or other parameters. A clinical protocol, as first suggested from Marmarou et al. [1] should be set up in order to thoroughly investigate and treat post-traumatic ventriculomegaly.

## Data Availability

Unfortunately, we did not have appropriate consent from our patients for sharing their data and, given the retrospective nature of our study, long recruitment time and no risk to anonymity, it has been impossible to obtain this.

## Abbreviations

AMP: Fundamental pulse amplitude of ICP
AMPb: AMP at baseline
AMP-P: AMP at plateau
CSF: Cerebrospinal fluid
dAMP: Difference between AMP plateau and AMP baseline
DC: Decompressive craniectomy
EVD: External ventricular drain
FHW: Frontal horn width
FOHR: Frontal-occipital horn ratio
ICP: Intracranial pressure
ICPb: ICP at baseline
ICPp: ICP at plateau
iNPH: Idiopathic normal pressure hydrocephalus
PTH: Post-traumatic hydrocephalus
RAP: Compensatory reserve index correlation coefficient between ICP and AMP
RAPb: RAP at baseline
RAPinf: RAP during infusion
Rout: Resistance to CSF outflow [mmHg/ml/min]
TBI: Traumatic brain injury
